# Effectiveness of the adjunctive use of ozone and chlorhexidine in patients with chronic periodontitis

**DOI:** 10.1038/s41405-019-0025-9

**Published:** 2019-11-28

**Authors:** Kaveri Kranti Gandhi, Emil G. Cappetta, Rajdeep Pavaskar

**Affiliations:** 1 Rutgers School of Dental Medicine, Newark, NJ USA; 2 Department of Periodontology, Rutgers School of Dental Medicine, Newark, NJ USA; 3 Goa Dental College and Hospital, Goa, India

**Keywords:** Periodontitis, Periodontitis

## Abstract

**Background:**

Scaling and root planning (SRP) is the gold standard approach for treatment of chronic periodontitis but used alone it may not be effective in removing periodontal pathogens from sites where access is poor.

**Objective:**

To evaluate and compare the clinical and microbiological efficacy of ozone and chlorhexidine (CHX) as an adjunct to SRP in patients with chronic periodontitis.

**Methods:**

Twenty-five patients with generalized moderate to severe chronic periodontitis with presence of at least one site in each quadrant with a probing depth ≥5 mm were recruited. In a split mouth study design, two quadrants were randomly allocated to the SRP and ozone therapy and the remaining two quadrants to SRP and CHX therapy. Plaque index (PI), Gingival index (GI), probing depth (PD), clinical attachment loss (CAL) were assessed. Subgingival plaque samples were obtained for assessment of *Aggregatibacter actinomycetemcomitans* (Aa) and *Porphyromonas gingivalis* (Pg).

**Results:**

Both groups demonstrated significant intragroup reduction in PI, GI, PD, CAL, Pg count and Aa count from baseline to 3 months follow-up. There were no significant differences between two groups for any of the parameters.

**Conclusion:**

Ozonated olive oil can be used as an adjunctive subgingival irrigant in patients with chronic periodontitis.

## Introduction

Periodontal infection is initiated by specific invasive oral pathogens that colonize dental plaque biofilms on tooth surface, and host immune response to inflammation plays a central role in disease pathogenesis.^1^ SRP is the most widely used technique in periodontal therapy. Studies related to root planing^2,3^ have shown that complete removal of calculus is usually difficult and incomplete removal of subgingival plaque has been stated to be equal to no plaque control at all.^4^ It has been observed that complete removal of plaque and calculus is more difficult in deep^4,5^ than in shallow pockets.

Ozone is a naturally occurring gas created from three oxygen atoms. It is a powerful oxidizing agent with a high antimicrobial power against oral pathogens.^6,7^ The antibacterial action of ozone is related to its reacting capacity with lipid double bonds, thus leading to bacterial wall lysis and bacterial cell content extravasation. By entering the cell, ozone promotes oxidation of nucleic and amino acids; and cell lysis depends on the extent of these reactions.^8^ It also inactivates viruses by diffusing through the protein coat into the nucleic acid core, resulting in damage of the viral nucleic acid. The antimicrobial action of ozone is nonspecific and selective to microbial cells; it does not damage human body cells because of their major antioxidative ability.^9^ Nagayoshi et al. examined the effect of ozonated water on oral microorganisms and dental plaque. Dental plaque samples were treated with 4 ml of ozonated water for 10 s. They observed that ozonated water was effective for killing gram-positive and gram-negative oral microorganisms and oral *Candida Albicans*.^10^ Ozone also seems to have immune-stimulating, antihypoxic and biosynthetic effects on human body.^11^ Ozone influences cellular and humoral immune system, stimulates proliferation of immunocompetent cells and synthesis of immunoglobulins, activates function of macrophages and increases sensitivity of microorganisms to phagocytosis. It improves the metabolism of inflamed tissues by increasing their oxygenation and reducing local inflammatory processes. It also activates mechanisms of protein synthesis, increases number of ribosomes and mitochondria in the cells. These changes on the cellular level explain the elevation of functional activity and regeneration potential of tissues and organs. Because of its properties, the use of ozone is currently being discussed in prevention of dental caries in fissures of the first permanent molars in children, in prepared cavity, after tooth extraction, in case of post-extraction complications, in patients with chronic gingivitis, periodontitis and periodontal abscesses, herpes labialis, purulent periodontitis, dentition difficilis, etc.^12^

Chlorhexidine is one of the most widely used oral antimicrobial agents and is available in different formulations. The substance is known to have good substantivity and at high concentrations (0.12% or more) is bactericidal, causing a lethal damage to the bacterial membrane, being active on both gram-negative and gram-positive bacteria. The common side effects associated with the use of CHX are increase in staining of teeth and other oral surfaces, an increase in calculus formation, and an alteration in taste perception. Currently, only limited data are available from controlled clinical trials comparing the efficacy of ozone therapy to the gold standard CHX. Therefore, the aim of this study was to evaluate and compare the effects of subgingival application of ozonated olive oil and 0.2% CHX as an adjunct to SRP in patients with chronic periodontitis. The objectives of the study were: (1) To evaluate and compare the effects of subgingival irrigation with CHX and ozonated olive oil on clinical parameters—PI, GI, PD and CAL. (2) To assess and compare the effect of CHX and ozonated olive oil on Pg and Aa counts by using bacterial culture method. The protocol was approved by the author’s institutional review committee for human subjects and the study was conducted in accordance with the Helsinki Declaration as revised in 2013.

## Materials and methods

### Patient selection and study design

A total of 25 patients, 30–60 years of age suffering from generalized moderate to severe chronic periodontitis, with the presence of at least one site in each quadrant with a probing depth ≥5 mm were selected. Patients with history of any systemic diseases, smokers, chronic alcoholics, pregnant and lactating women, patients who had received any periodontal therapy in last 6 months, patients with the history of use of any oral rinse or antibiotic therapy in last 6 months were excluded from the study. A randomized, double-blind, split mouth study design was performed. Randomization was carried out using computer generated random numbers. All treatment procedures were performed by a single periodontist. Clinical measurements were recorded and subgingival plaque sampling was carried out in all patients by another examiner. The following clinical parameters were recorded in all patients: PI according to Silness, P. and Loe, H. (1964); GI according to by Loe H and Silness J (1963); PD—It was measured from base of the pocket to the gingival margin at mesiobuccal, midbuccal, distobuccal and lingual surfaces using UNC-15 probe; CAL—It was measured from base of the pocket to the cementoenamel junction at mesiobuccal, midbuccal, distobuccal and lingual surfaces using UNC-15 probe. For microbiological evaluation, pooled subgingival plaque samples were obtained from all sites in each group with PD ≥ 5 mm at baseline. These sites were isolated with cotton rolls, following which supragingival plaque was removed using a sterile hand scaler and cotton gauge, to prevent any contamination of the samples. Subgingival plaque samples were obtained using a sterile gracey curette and sent for microbiological examination in a sterile container containing RTF (reduced transport fluid).

Informed consent was obtained from each patient after explaining the aims and objectives of the study. Clinical parameters were recorded, and subgingival plaque samples were collected at baseline before SRP and 3 months after the treatment.

### Treatment procedure

SRP was carried out in all patients using ultrasonic and hand instruments in two visits. In the first appointment, each patient received full mouth supragingival scaling with a piezoelectric handpiece (EMS). After 1 week, on the second appointment, full mouth subgingival SRP was done using both Gracey curettes (Hu-Friedy) and piezoelectric handpiece under local anesthesia of 2% lidocaine with 1:100,000 epinephrine. Following which, two quadrants were assigned to the CHX group and the remaining two quadrants were assigned to the ozonated olive oil group. All sites with probing depth ≥5 mm were isolated carefully with cotton rolls and thoroughly dried and CHX (0.2%) or ozonated olive oil was applied carefully subgingivally with the help of a disposable 2 ml plastic syringe and a 28-gauge needle. Patients were instructed not to eat, drink or rinse for at least 30 min. Subgingival application of ozonated olive oil and chlorhexidine was performed immediately after SRP on the second visit and was repeated 2 weeks after SRP.

### Power calculation and statistical analysis

Using G* Power 3.1.9.4 software the sample size was estimated to be 20. A sample size of 25 was recruited to compensate for the possible dropouts during the study. The sample size estimation before the start of the study considered beta error to be 20%. Post-hoc power analysis done after completion using G* Power 3.1.9.4 software suggested that the power achieved in the present study is 100%. Statistical analysis was performed using the statistical software SPSS version 20. For statistical analysis only sites with PD ≥ 5 mm at baseline were included in the analysis. Intragroup differences in clinical and microbiological parameters were analyzed by paired *t* test whereas the intergroup differences were analyzed using unpaired *t* test. Statistical significance was set at 95% probability level (*P* < 0.05).

## Results

All patients attended the third month follow-up appointment. No discomfort or side effects were reported by any of the patients. At baseline examination, there were no statistically significant differences between the groups with regard to any of the recorded parameters. Both the groups demonstrated significant intragroup reduction in PI, GI, PD, CAL, Pg count and Aa count from baseline to 3 months follow-up. However, on intergroup comparison, no statistically significant differences were found between the CHX and ozonated olive oil groups regarding any of the clinical and microbiological parameters at the follow-up visit (Tables 1–6).

**Table 1:** Comparison of mean values of Plaque Index

Groups	Baseline	At 3 mo.	Difference	*P* value
Ozonated olive oil	1.90 ± 0.12	0.66 ± 0.14	1.74 ± 0.21	0.00^a^
Chlorhexidine	1.86 ± 0.17	0.62 ± 0.21	1.24 ± 0.33	0.00^a^
Difference	0.04	0.04		
*P*	0.25	0.28		

^a^Represents statistically significant difference

**Table 2:** Comparison of mean values of Gingival Index

Groups	Baseline	At 3 mo.	Difference	*P* value
Ozonated olive oil	1.87 ± 0.12	0.79 ± 0.12	1.07 ± 0.19	0.00^a^
Chlorhexidine	1.88 ± 0.13	0.81 ± 0.12	1.07 ± 0.19	0.00^a^
Difference	0.01	0.02		
*P*	0.66	0.79		

^a^Represents statistically significant difference

**Table 3:** Comparison of mean values of probing depth

Groups	Baseline	At 3 mo.	Difference	*P* value
Ozonated olive oil	5.47 ± 0.32	3.01 ± 0.40	2.48 ± 0.23	0.00^a^
Chlorhexidine	5.41 ± 0.32	2.78 ± 0.47	2.6 ± 0.54	0.00^a^
Difference	0.06	0.23		
*P*	0.96	0.68		

^a^Represents statistically significant difference

**Table 4:** Comparison of mean values of clinical attachment loss

Groups	Baseline	At 3 mo.	Difference	*P* value
Ozonated olive oil	5.02 ± 0.37	2.92 ± 0.43	2.10 ± 0.27	0.00^a^
Chlorhexidine	4.78 ± 0.37	2.63 ± 0.40	2.12 ± 0.48	0.00^a^
Difference	0.06	0.23		
*P*	0.96	0.68		

^a^Represents statistically significant difference

**Table 5:** Comparison of mean values of bacterial counts of *Porphyromonas gingivalis*

Irrigants	Baseline	At 3 mo.	Difference	*P* value
Ozonated olive oil	77.80 ± 38.52	0.90 ± 0.87	76.90 ± 38.09	0.00^a^
Chlorhexidine	82 ± 33.35	0.90 ± 0.87	81.10 ± 32.85	0.00^a^
Difference	4.20	0.00		
*P*	0.75	1.00		

^a^Represents statistically significant difference

**Table 6:** Comparison of mean values of bacterial counts of *Aggregatibacter Actinomycetemcomitans*

Groups	Baseline	At 3 mo.	Difference	*P* value
Ozonated olive oil	19.00 ± 15.59	0.20 ± 0.42	18.80 ± 15.33	0.004^a^
Chlorhexidine	22.00 ± 12.97	0.30 ± 0.67	21.70 ± 12.63	0.00^a^
Difference	3.00	0.10		
*P*	0.55	0.33		

^a^Represents statistically significant difference

## Discussion

The mechanical removal of plaque and calculus and the adjunctive use of antibiotics and antiseptics have been the conventional methods for periodontal therapy. The powerful antimicrobial action of ozone, with its ability to modulate the immune response makes it a potential therapeutic agent in the treatment of multifactorial periodontal disease. Additionally, ozone is known to activate angiogenesis. This process is brought about by the secretion of vasodilators like nitric oxide (NO). Nitric oxide causes vasodilation of arterioles and releases growth factors like vascular endothelial growth factor (VEGF) which helps in angiogenesis.^13^ Topical administration of ozone can be performed in gaseous form through an open system or through a suction or as ozonated water and ozonated oil. In this study, ozonated olive oil was selected over ozonated water because the application of oil has been found to provide a long stay in the oral cavity, adequate drug penetration, high efficacy and acceptability.^14^ A split-mouth design was used to eliminate patient-specific conditions and allow the comparison of both treatment methods under similar conditions.

The use of ozone therapy in treatment of chronic periodontitis has been evaluated by many studies. While some studies showed the additional benefit of using ozone therapy,^15–19^ others demonstrated that the adjunctive use of ozone therapy provided no additional benefit.^20–22^ In our study, no significant difference was found in the efficacy of ozonated olive oil and CHX in improving the PI, GI, PD, clinical attachment levels and reducing the Pg and Aa. However, our results differ from those obtained by Kshitish and Laxman.^16^ They observed a higher percentage of reduction in PI, GI, and bleeding index and Aa using ozone as compared to using CHX. Another study^17^ that compared the effectiveness of ozone with that of the CHX, against periodontal microorganisms reported no significant differences in the effectiveness of aqueous ozone or gaseous ozone compared with 2% CHX but they were more effective than 0.2% CHX.

Ramzy et al.^18^ reported a highly significant improvement in clinical parameters and bacterial count in quadrants treated by SRP together with ozone application in comparison to SRP alone in patients with aggressive periodontitis. On the other hand, irrigation with ozonated water as an adjunctive therapy to SRP produced no statistically significant benefit compared with SRP plus distilled water irrigation in the study by Al Habashneh et al.^20^ However, both these studies did not test the efficacy of CHX in comparison to ozone. Müller et al.^22^ compared the influence of ozone gas with photodynamic therapy and antiseptic agents 2% CHX, 0.5 and 5% hypocholrate solutions on a multispecies oral biofilm in vitro*. Actinomyces naeslundii, Veillonelladispar, Fusobacterium nucleatum, Streptococcussobrinus, Streptococcus oralis and C. albicans* were studied. They concluded that the matrix-embedded microbial populations in biofilm are well protected towards antimicrobial agents. Only 5% hypochlorate solution was able to eliminate all bacteria effectively. Usage of gasiform ozone or PDT was not able to reduce bacteria in the biofilm. In a study^19^ that evaluated the antimicrobial efficacy of Er:YAG laser and topical gaseous ozone, the authors found that ozone has antimicrobial effect equivalent to that of the Er:YAG laser.

## Conclusion

The adjunctive use of ozonated olive oil in the treatment of chronic periodontitis significantly improves clinical and microbiological results and is equally effective as chlorhexidine, and at the same time is free from adverse effects.

## References

[1] Saini R, Marawar PP, Shete S, Saini S (2009). Periodontitis, a true infection. J. Glob. Infect. Dis..

[2] Jones SJ, Lozdan J, Boyde (1972). Tooth surfaces treated in situ with periodontal instruments. Scanning electron microscopic studies. Dent. J..

[3] Jones W, O'Leary TJ (1978). The effectiveness of in vivo root planing in removing bacterial endotoxin from the roots of periodontally involved teeth. J. Periodontol..

[4] Waerhaug J (1978). Healing of the dento-epithelial junction following subgingival plaque control. II. As observed on extracted teeth. J. Periodontol..

[5] Lovdal A, Arno A, Schei O, Waerhaug J (1961). Combined effect of subgingival scaling and controlled oral hygiene on the incidence of gingivitis. Acta Odontol. Scand..

[6] Huth KC (2009). Effectiveness of ozone against endodontopathogenic microorganisms in a root canal biofilm model. Int Endod. J..

[7] Baysan A, Lynch E (2004). Effect of ozone on the oral microbiota and clinical severity of primary root caries. Am. J. Dent..

[8] Atiyeh BS, Dibo SA, Hayek SN (2009). Wound cleansing, topical antiseptics and wound healing. Int Wound J..

[9] Gupta G, Mansi B (2012). Ozone therapy in periodontics. J. Med. Life.

[10] Nagayoshi M (2004). Efficacy of ozone on survival and permeability of oral microorganisms. Oral Microbiol. Immunol..

[11] Seidler V, Linetskiy I, Hubalkova H (2008). Ozone and its usage in general medicine and dentistry. A review article. Prague Med. Report.

[12] Srikanth A, Sathish M, Sri Harsha AV (2013). Application of ozone in the treatment of periodontal disease. J. Pharm. Bioallied Sci..

[13] Vinutha RS, Lakshmanan R (2014). Ozone and its role in periodontal therapy—a review IOSR. J. Dent. Med. Sci. (IOSR-JDMS).

[14] Jain N (2008). Recent approaches for treatment of periodontitis. Drug Discov. Today.

[15] Hauser-Gerspach I (2012). Influence of gaseous ozone in peri-implantitis: bactericidal efficacy and cellular response. An in vitro study using titanium and zirconia. Clin. Oral Investig..

[16] Kshitish D, Laxman VK (2010). The use of ozonated water and 0.2% chlorhexidine in the treatment of periodontitis patients: a clinical and microbiologic study. Indian J. Dent. Res..

[17] Huth KC (2011). Effectiveness of ozone against periodontal pathogenic microorganisms. Eur. J. Oral Sci..

[18] Ramzy MI, Gomaa HE, Mostafa MI (2005). Management of aggressive periodontitis using ozonized water. Egypt Med. J. Nrc..

[19] Yilmaz S (2013). Evaluation of the clinical and antimicrobial effects of the Er:YAG laser or topical gaseous ozone as adjuncts to initial periodontal therapy. Photomed. Laser Surg..

[20] Al Habashneh R, Alsalman W, Khader Y (2015). Ozone as an adjunct to conventional nonsurgical therapy in chronic periodontitis: a randomized controlled clinical trial. J. Periodontal Res..

[21] Skurska A (2010). Evaluation of the influence of ozonotherapy on the clinical parameters and MMP levels in patients with chronic and aggressive periodontitis. Adv. Med. Sci..

[22] Müller P, Guggenheim B, Schmidlin PR (2007). Efficacy of gasiform ozone and photodynamic therapy on a multispecies oral biofilm in vitro. Eur. J. Oral Sci..

